# ASM incorporates Imagetwin to address image duplication and preserve scientific accuracy

**DOI:** 10.1128/mbio.01990-25

**Published:** 2025-08-25

**Authors:** Aashi P. Chaturvedi, Alicea Hibbard, Chad Nelson, Arturo Casadevall, Amy L. Kullas

**Affiliations:** 1 Department of Ethics and Integrity, Journals, American Society for Microbiology 11003, Washington, DC, USA; 2 Department of Molecular Microbiology and Immunology, Johns Hopkins School of Public Health 51561 , Baltimore, Maryland, USA; Albert Einstein College of Medicine, Bronx, New York, USA

**Keywords:** image duplication, AI tools, image integrity, scientific integrity, Imagetwin

## Abstract

Image duplication in scientific articles—accidental or intentional—undermines trust in research, authors, institutions, and publishers. Duplications not only cast doubt on researchers’ scientific rigor, but they also raise concerns about potential misconduct, jeopardizing careers and even calling into question the effectiveness of the peer review process and editorial oversight. Ultimately, these issues erode confidence in the published study and, more broadly, the entire scientific community. However, when combined with expert in-house staff verification and artificial intelligence-based tools like Imagetwin, Proofig, etc., publishers can detect potential image duplications before publication and strengthen the integrity of the scientific record. In 2023, the American Society for Microbiology (ASM) Journals program integrated Imagetwin into its editorial workflow and conducted a 1-year pilot study. Here, we present key findings and highlight how ASM Journals refined its processes to incorporate image duplication screening earlier in the manuscript lifecycle. The pilot identified image duplications prior to publication in 3.9% of accepted, eligible manuscripts screened with Imagetwin. Most image concerns were unintentional and readily resolved. Of the 2,627 accepted manuscripts screened during the pilot, acceptance was revoked for six (0.23%) due to unresolved issues. It is now a key component of the routine ethics checks performed by ASM journals.

## PERSPECTIVE

A June 2016 study revealed a startling finding: approximately 1 in 25 biomedical research papers contained inappropriately duplicated images. In this study, image duplication refers to the reuse of identical or overlapping images or portions of an image within a manuscript or between the manuscript and previously published articles. Such duplications, whether within a single figure, across figures, or between publications, can raise concerns about data integrity. Up to 3.8% of published papers contained at least one problematic figure—half displaying signs of intentional manipulation. The presence of even a single problematic figure can warrant significant corrective actions, including author corrections or retraction of the article. The authors further noted that such concerns are rising (1). Several factors influence the likelihood of image manipulation in scientific research: cultural academic pressure to publish, cash-based publication incentives, and inconsistent policies on handling scientific misconduct. Paper mills are known to use previously published or falsified images and even AI-generated figures, which is a growing concern. These problems are extremely hard to detect without the assistance of equally powerful screening tools. Pre-publication image screening significantly reduces the staff time required to address issues, underscoring the importance of early detection in minimizing resource-intensive post-publication corrections or retractions (2).

## IMAGE DUPLICATION: A GROWING CONCERN

This highlights the global importance of promoting research and data integrity through investment, such as screening tools, that address the growing challenges posed by image fraud, enhancement, and genuine errors in scientific research (3). In recent years, journals have explored AI-based software to automatically detect and alert journals to image duplication concerns but with limited success (4). Since images are integral representations of underlying data and methods, particularly in fields like microbiology, ensuring their scientific integrity is essential to ASM and the larger STEM field. Since 2013, ASM has relied on dedicated image specialist staffing to utilize various Office of Research Integrity (ORI) and Adobe tools to scan accepted manuscripts for image splicing, manipulation, and non-uniform enhancements (selective modifications of only portions of an image). Until recently, unless an image was brought to the journal’s attention by a reviewer, editor, or sleuth, accurate detection of duplicate images was infeasible.

To address this gap, in 2023, ASM initiated a pilot study using the AI-based software, Imagetwin (https://imagetwin.ai/), launched in 2022. Its compatibility with ASM’s workflow and abilities to automatically compare pixel-level similarities, cross-reference images with a growing database of published content, and flag duplicates in a user-friendly manner became a valuable addition to our scientific integrity processes.

It is important to note that there are limitations to the software. Not all images can be screened by Imagetwin. During the pilot study, concerns were identified exclusively in halftone images, such as western blot images, microscopy images, and photographs. However, it could not detect issues with line art, sketches, or graphs. Human training, review, and oversight are essential to validate output and filter out false positives. This article highlights key findings from the pilot study and the adjustments made by ASM Journals for effective workflow integration. When combined with expert in-house verification, this screening tool helps detect and address image duplication concerns more efficiently, consistently, and prior to publication.

## STARTING SMALL: A SINGLE JOURNAL TITLE

The initial month-long pilot program began with ASM’s sound science journal, *Microbiology Spectrum*. During this initial period, partial or complete image duplications were identified in 16 accepted manuscripts; some featured multiple instances of duplication. While most concerns were resolved by the authors revising figures or adding appropriate attribution, three cases proved more complex. In one instance, authors were asked to repeat the entire internally-controlled experiment, and the manuscript was subjected to further editorial review. Another required extensive image corrections, and the manuscript was revised and resubmitted. In the third case, an insufficient explanation from the authors meant acceptance was revoked. Recognizing the utility of the screening tool, we quickly expanded the Imagetwin pilot to all ASM journals in March 2023.

**Fig 1:**
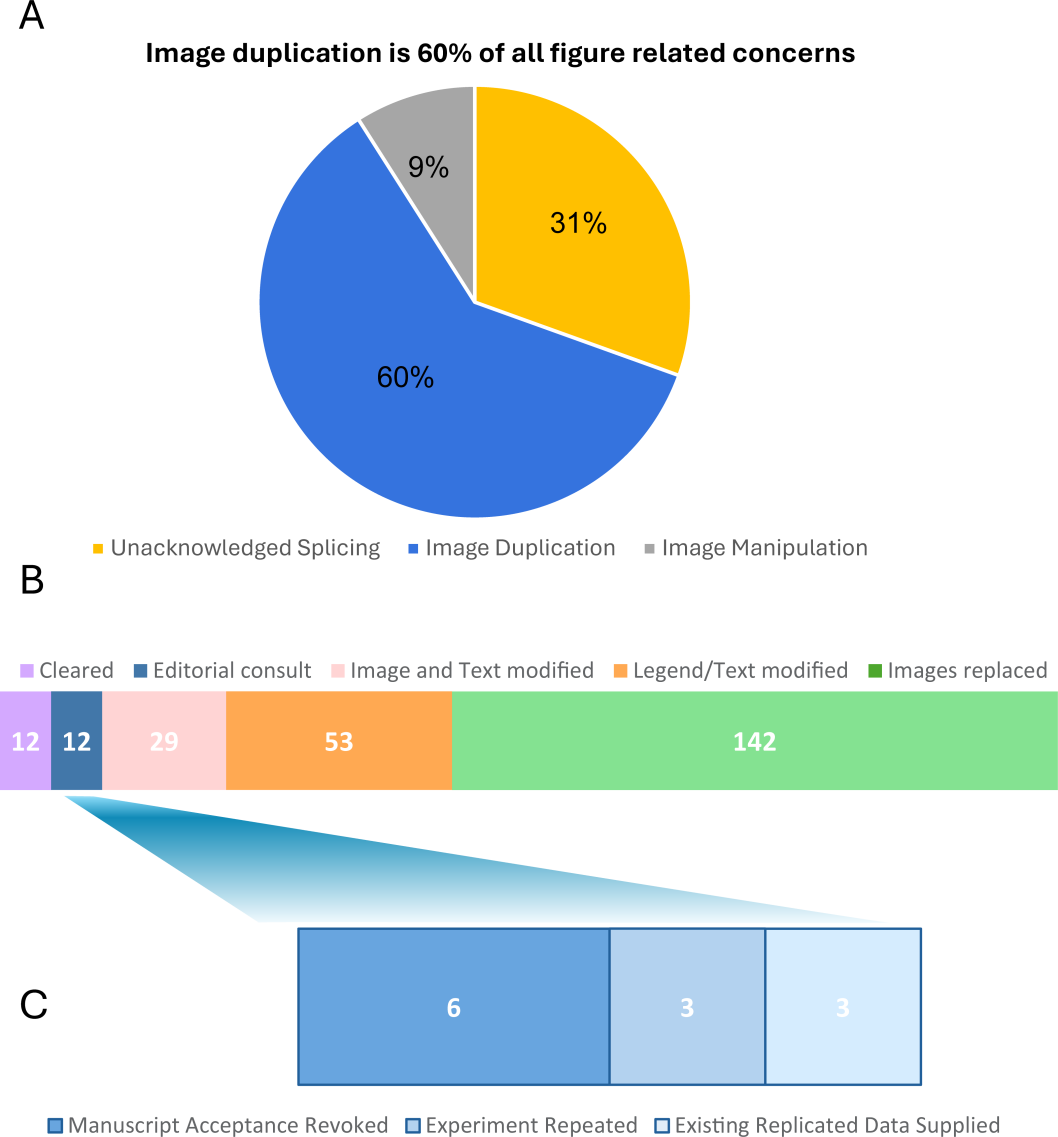
ASM figure pre-screening workflow can identify image duplication concerns using Imagetwin and address them before publication. (**A**) Image duplication represents two-thirds of figure-related concerns in post-acceptance manuscripts. (**B**) All image duplication concerns were resolved following our policies and procedures in accordance with ethical guidelines by modifying the figures and/or text. (**C**) Complex image duplication concerns were resolved through editorial consultations, which sometimes led to revoking acceptance decision for the manuscript.

Data are presented from our full pilot study of all 15 journals over a 12-month period from 1 March 2023 to 1 March 2024. Figure 1A shows that after applying Imagetwin screening to accepted manuscripts, a substantial number (410 out of 2,627) exhibited image-related concerns. Out of these, nearly 60% (248 out of 410) of figure-related issues were due to image duplication. Of these duplications, approximately 5.3% involved images duplicated from previously published articles. The remaining image integrity issues were due to unacknowledged splicing in 125 manuscripts and non-uniform image enhancements in 37 manuscripts.

## FIGURE SCREENING WORKFLOW DURING THE PILOT PROGRAM

Over the past 10 years, ASM has averaged approximately 6,300 accepted manuscripts from nearly 17,500 submissions to its journals each year. Mindful of volume and resources, routine prescreening of figures was conducted after manuscript acceptance. This post-acceptance screening used tools from ORI and Adobe to detect the presence of splicing, merging, non-uniform image enhancements, and image manipulation in figures. As mentioned earlier, at the time, Imagetwin detected duplication only in halftone images. During the pilot project, all accepted manuscripts with relevant images were automatically screened with Imagetwin, in addition to routine checks.

To minimize false positives, a visual inspection by the image specialist followed, and any remaining concerns were further verified using the “Difference” function in Adobe Photoshop. In practice, two images are placed on separate layers and the difference mode compares the color values of the two layers. When layers are identical, a completely black image results because the subtraction of identical pixels results in zero, appearing as black. When the layers are not identical, the Difference mode creates an inverted or high-contrast version of the image based on the pixel differences between layers. This additional verification minimized false positives and provided confidence in the Imagetwin findings. Only issues that were flagged in all stages of screening—Imagetwin scan, visual inspection, and Adobe Difference function analysis—were included in the official “figure concerns report.”

This report was subsequently shared with the ASM Journals ethics department for further review. The ethics team would contact the authors for clarification and, when necessary, request the original underlying data. In most cases, authors were unaware of these issues and appreciated the identification prior to publication. Manuscripts were held until all concerns were addressed. Upon resolution, manuscripts were allowed to proceed to publication and the authors were informed. The findings, along with relevant details, such as manuscript information, the nature of the concern, and its resolution, were documented for record-keeping.

## CRITICAL INSIGHTS

The most common reasons for image duplication were unintentional duplicate use of placeholder images or intentional re-use of control images for large experiments. Other reasons included inappropriate figure data storage, mislabeled folders, and human error during figure assembly.

Through interaction with the authors, we also discovered knowledge gaps in publishing policies and their application. For instance, authors might be aware that reusing figures from previous publications requires acknowledgment. However, they may fail to cite the original article appropriately or obtain the necessary copyright permissions. Similarly, authors may not know that reusing the same control image within an article needs to be transparently acknowledged. Scholarly publishers should focus on educating authors and ensuring compliance to prevent easily rectifiable and preventable situations. Presentations at editorial meetings, Q&A sessions with editors, and webinars with authors, reviewers, and editors were all highly effective.

Addressing concerns in accordance with the authors' responses was an invaluable learning experience. When authors provided original data or satisfactory explanations, they were permitted to either replace duplicate images with the correct representative images or acknowledge the duplication in the figure legend. Additional detailed text modifications in the Materials and Methods section were requested as necessary. In some cases, both figure and text modifications were required to enhance clarity for the reader. Figure 1B illustrates the distribution of manuscript resolutions.

When responses were unsatisfactory or authors could not provide original underlying data, editorial consultations were crucial (12 out of 410 concerns). The consultation process varies by journal but generally involves the ethics team and all relevant parties from the journal. The ethics team provided necessary materials and policy information while coordinating author communication. Managing editors were kept informed and offered their expertise, while research-active handling editors, senior editors (when available), and the editor in chief consulted for subject matter expertise and to ensure alignment with the journal’s expectations. This approach also allowed consistency across ASM Journals in resolving image duplication concerns. These editorial consultations addressed issues such as excessive duplications in manuscripts, duplicated sections in images, or insufficient explanations from authors. In 3 out of 12 editorial consults, authors were requested to repeat new internally controlled experiments to verify the data. Authors were allowed to present data from replicate experiments in 3 out of 12 consults. Replacing existing data with revised figures from replicate or newly repeated experiments did require additional editorial review and approval which added more time to the manuscript’s lifecycle.

## HARD LESSONS

In six out of 12 editorial consults, manuscript acceptance was revoked because authors did not provide satisfactory explanations or original data were not made available (Fig. 1C). Acceptance revocation was a difficult decision and was not made lightly, but it allowed ASM to emphasize to our authors the critical importance of proper data processing, storage, and management. Additionally, it provided an opportunity to inform authors about ASM Journals’ policies on appropriate handling of images for figure generation. Although rare, these instances underscored the need to modify our workflow. By incorporating Imagetwin screening before manuscript acceptance, we could prevent such revocations, thereby saving significant time and resources for authors, journal staff, and editors.

Upholding research integrity through decisions, such as requesting experimental repetition or revoking manuscript acceptance, required careful judgment and came with significant operational demands. On average, addressing image duplication involved approximately 14 email exchanges among authors, ethics team, journal staff, and editors. In more complex cases, particularly those involving repeated experiments or altered editorial outcomes, this number increased to as many as 42 emails, reflecting the added burden of coordinating timelines, reviewing revised data, and updating internal workflows. These efforts underscore the considerable time and staff resources necessary to support such integrity-driven processes.

## IMPROVING AUTHOR EXPERIENCE

After completion of the pilot program, Imagetwin screening was moved to the revision or resubmission stage where other ethics checks, such as those for text similarity, etc., are performed routinely. As in the pilot process, flagged images are verified to rule out false positives. Confirmed image integrity issues are reported to the authors to seek clarification and resolution. If the response is unsatisfactory or ambiguous, original underlying data are missing, or additional concerns arise during this process, then editors and journals can consider rejecting the manuscript at this stage. If all image integrity concerns are resolved, the journal can proceed with these manuscripts with greater faith in the data.

Imagetwin integration in our routine prescreening process has proven effective in detecting image duplication issues before publication. We found that about 3.9% of accepted manuscripts (248 out of 6416) had image duplication problems, aligning with the 3.8% duplications reported in the 2016 *mBio* study (1). This indicates that the rate of image duplication, at least in this data set, has not increased, and we are identifying a significant number of image duplications prior to publication.

This proactive approach helps reduce post-publication image integrity critiques and enhances the ability to address these concerns effectively in pre-publication manuscripts. Additionally, Imagetwin is used to verify existing post-publication critiques about potential image duplications in ASM Journals’ articles. As automated tools become more precise and their database grows, their capacity to detect duplication across manuscripts, open-access publications, and preprints is expected to improve significantly.

Early in 2025, *Science* announced their implementation of a different image integrity screening tool called Proofig (https://www.proofig.com/) to facilitate detection of image duplication and manipulation (5). This shows an increased industry interest in proactive image integrity issue detection.

## BOTTOM LINE CONSIDERATIONS

Cost is an important consideration for journals and publishers when adopting new tools, but ASM’s 1-year pilot program clearly demonstrated that investing in these tools is worthwhile. Our tracking shows that resolving figure concerns post-publication can take up to 10 h of staff time, while pre-publication resolution averages just 1.5 h. This represents substantial savings in resources and time for both publishers and authors. We recommend other publishers, especially those handling halftone images like microscopy and gels, consider implementing similar solutions. Detecting problematic or reused images is challenging, particularly with the rise of paper mills. Moreover, current tools for detecting AI-generated or AI-modified images are still under development and lack robustness and consistent reliability. Investing in image integrity tools is a crucial step towards strengthening the peer review process and ensuring the integrity of scientific publications.
